# A LAMP (loop‐mediated isothermal amplification) test for rapid identification of Khapra beetle ( *Trogoderma granarium* )

**DOI:** 10.1002/ps.6591

**Published:** 2021-08-18

**Authors:** Lea Rako, Arati Agarwal, Linda Semeraro, Adam Broadley, Brendan C Rodoni, Mark J Blacket

**Affiliations:** ^1^ Agriculture Victoria AgriBio Bundoora VIC Australia; ^2^ Department of Agriculture, Water and the Environment Science and Surveillance Group Canberra ACT Australia

**Keywords:** Khapra beetle LAMP assay, 18S LAMP control assay, field diagnostics, Dermestidae, Khapra beetle identification, loop‐mediated isothermal amplification (LAMP), *Trogoderma granarium*

## Abstract

**BACKGROUND:**

Khapra beetle (*Trogoderma granarium* Everts) is a significant pest of food products around the world, causing great losses of stored grain and produce, with export restrictions imposed on countries with established beetle populations. Khapra beetle is a high‐priority exotic invertebrate pest in many countries requiring a rapid quarantine/biosecurity response when incursions occur. To address this, we developed a novel Khapra LAMP (loop‐mediated isothermal amplification) assay using a portable real‐time fluorometer and an additional 18S ribosomal DNA (18S) insect control LAMP assay for confirmation of the presence of insect DNA. Both LAMP tests can be performed either in a portable real‐time fluorometer or using simple, visual colorimetric technique.

**RESULTS:**

Both the Khapra and 18S LAMP tests amplify positive samples within ≤ 25 min, with an anneal derivative temperature of 77.7 ± 0.7 °C for Khapra LAMP test and 88.0 ± 1.0 °C for 18S. The new Khapra LAMP assay is sensitive to very low levels of DNA (1.02 × 10^**−**6^ ng μL^−1^). Additionally, we developed a gBlock double stranded DNA fragment for use as positive Khapra control with a different anneal derivative of 80 °C. Both assays are simple to use in the field and are capable of amplifying DNA from target beetles, even when samples are partially degraded which is typically found during surveillance activities. By screening a broad panel of Dermestidae species we demonstrate that our new assay is species‐specific, with no detections of false positives. Also, we evaluated multiple DNA extraction methods, with both QuickExtract and HotSHOT extraction methods proving suitable for in‐field use.

**CONCLUSION:**

The novel Khapra and 18S LAMP assays should improve speed, accuracy and confidence of detection of Khapra beetle at incursion points and aid rapid biosecurity responses in any country affected, especially as the assays described here are portable and easy to implement in the field conditions where resources are limited. © 2021 The Authors. *Pest Management Science* published by John Wiley & Sons Ltd on behalf of Society of Chemical Industry.

## Graphical abstract

- We developed Khapra LAMP (loop‐mediated isothermal amplification) test for use in a portable fluorometer (amplifies positives within 25 min, anneal temperature 77.7 °C), with a gBlock double stranded DNA fragment as a positive control.
- The assay is sensitive to very low levels of DNA (1.02 × 10^−6^ ng μL^−1^) and capable of amplifying DNA from target beetles, even when samples are partially degraded.
- Our assay can be run as an alternative LAMP colorimetric detection method, that can be performed using only a heat block.
- Multiple DNA extraction methods were also tested, with both the QuickExtract and HotSHOT extraction methods proving suitable for in‐field use.

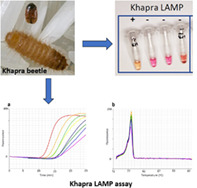

## 1 INTRODUCTION

Khapra beetle (*Trogoderma granarium* Everts) is a member of a large family of beetles (Coleoptera: Dermestidae) with approximately 1000 described species and 50 genera,^1^ including many well‐known pests, however the majority are not considered economically important.^1^, ^2^ Khapra beetle is a serious pest of stored grain and dry foodstuffs of great importance worldwide and can cause loss of produce of up to 75% by feeding and contamination from larval castings, which are very difficult to remove and clean. In optimal environmental conditions this pest can have more than ten generations per year with the complete lifecycle varying between 26 to 220 days, depending on conditions.^3^, ^4^, ^5^ Populations can persist for prolonged periods, further infesting other material and increasing the likelihood of contamination of produce by the fungus *Aspergillus flavus*.^6^ Additional problems are caused by export restrictions imposed on the countries with established Khapra beetle populations. *Trogoderma granarium* has very limited ability to spread without human help as adults do not fly, therefore their long‐distance dispersal is primarily assisted by human trade and traffic of goods.

Adult dermestids are often very similar morphologically, requiring specialized entomological knowledge to distinguish between them, with larvae being even more morphologically obscure.^5^, ^7^, ^8^ Often, due to highly specialized morphological knowledge required to identify insect species they can be misidentified in bulk samples,^9^ resulting in false permanent record of the presence of an insect that is not correctly named. For example, such a misidentification has previously occurred, and Australia was erroneously listed as a ‘Khapra beetle’ country in the late 1940s. It took over 15 years of lobbying and publication effort to have this stigma removed.^10^ Today, Australia is Khapra beetle free,^11^, ^12^, ^13^ but intercepted specimens at the nations border are being increasingly encountered due to the increased traffic and trade of goods around the world.^14^, ^15^ This places Khapra beetle amongst the highest priority pests listed in Australia^13^ requiring reliable rapid biosecurity identification protocols with a need to confidently differentiate between invasive Khapra beetle and numerous native Dermestidae.

Globally, in addition to morphological tools for Khapra beetle identification,^5^, ^8^ there is an urgent need for field‐deployable, cost‐effective diagnostic tests for rapid pest detection that can be delivered in a timely manner, as current diagnostic technologies such as DNA barcoding,^16^ real‐time polymerase chain reaction (PCR)^17^, ^18^ require complex protocols, highly‐skilled technical staff, and are costly and difficult to undertake outside of the laboratory. LAMP, a DNA based assay developed for specific targets using loop‐mediated isothermal amplification method^19^, ^20^, ^21^ provides the capability to quickly train staff and implement field surveillance, enabling effective incursion response, containment and eradication programmes of invasive pests before they establish themselves in the new environment.^15^, ^22^ The ability to conduct field‐tests for pest identification rapidly and reliably further reduces the burden on national diagnostic laboratories^23^, ^24^ reducing the number of samples requiring specialist entomological analysis. By providing timely pest status the Khapra beetle LAMP assay will provide ‘evidence of absence’ of this priority pest to industry and support market access requirements for countries ‘free from Khapra beetle’.

The aims of this study were to: (i) Report on the development and optimization of a novel LAMP assay to identify Khapra beetle (*T. granarium)*; (ii) Assess the new assay in a portable real‐time fluorometer for reliability, specificity and sensitivity; (iii) Assess and compare the performance of a variety of DNA extraction methods, including methods suitable for in‐field use; (iv) Utilize a new insect‐specific 18S ribosomal DNA (18S) LAMP as a control assay to confirm the presence of insect DNA; (v) Design and evaluate a synthetic gBlock dsDNA fragment for use as a reliable Khapra DNA positive control in the LAMP assay; and (vi) Assess an alternative simple colorimetric method for both LAMP tests.

## 2 MATERIALS AND METHODS

### 2.1 Specimens examined

Specimens of native and exotic Dermestidae adults and larvae, other than Khapra, examined in this study were acquired through routine Dermestidae surveys conducted in the greater Melbourne (Australia) region, in 2018–2020 (*n* = 75), or from two Department of Agriculture, Water and the Environment (DAWE) biosecurity national border interceptions (*n* = 44), respectively (Table 1). In addition to the exotic, intercepted Khapra beetle, the other species tested in this study belonged largely to genera *Trogoderma*, *Anthrenus*, *Anthrenocerus*, *Attagenus*, *Dermestes*, *Orphinus*, *Reesa*, with a small number of additional taxa which remained undetermined, but are likely to be native Australian fauna (Table 1). Specimens were confirmed to the genus or species where possible by morphological means^5^, ^8^ and through DNA barcoding of the mitochondrial 16S locus.^17^ In the genus *Trogoderma*, 31 exotic *T. granarium*, one exotic *Trogoderma glabrum* Herbest, 30 locally collected *Trogoderma variabile* Ballion, and four additional *Trogoderma* species were tested (Table 1).

**Table 1:** Panel of Dermestidae species tested for the Khapra LAMP (loop‐mediated isothermal amplification) assay

Genus	Species	*n*	Lifestage	Specimen source	GenBank accession^†^	Minimum percentage sequence difference (Khapra)^†^	Khapra LAMP
*Trogoderma*	*Trogoderma granarium*	31	Adult/larval	DAWE, International Interception	MZ571636–MZ571637	0	Positive
*Trogoderma*	*Trogoderma glabrum*	1	Larval	DAWE, International Interception	MZ571638	5.5	Negative
*Trogoderma*	*Trogoderma variabile*	30	Adult	AgVic, Routine Dermestidae Survey	MZ571639	13.7	Negative
*Trogoderma*	*Trogoderma* sp.1	1	Adult	DAWE, International Interception	MZ571640	18.5	Negative
*Trogoderma*	*Trogoderma* sp.2	1	Adult	DAWE, International Interception	MZ571641	17.9	Negative
*Trogoderma*	*Trogoderma* sp.3	1	Adult	DAWE, International Interception	MZ571642	18.5	Negative
*Trogoderma* ^*^	*Trogoderma* sp.4	1	Adult	AgVic, Routine Dermestidae Survey	MZ571643	11.1	Negative
*Anthrenus*	*Anthrenus verbasci*	31	Larval	AgVic, Routine Dermestidae Survey	MZ571644	18.4	Negative
*Anthrenocerus*	*Anthrenocerus australis*	1	Larval	DAWE, International Interception	MZ571645	15.9	Negative
*Attegenus*	*Attegenus pellio*	1	Adult	DAWE, International Interception	MZ571646	20.3	Negative
*Dermestes*	*Dermestes lardarius*	1	Adult	DAWE, International Interception	MZ571647	26.8	Negative
*Dermestes*	*Dermestes maculatus*	1	Adult	DAWE, International Interception	MZ571648	24.5	Negative
*Dermestes*	*Dermestes peruvianus*	1	Adult	DAWE, International Interception	MZ571649	23.7	Negative
*Dermestes*	*Dermestes ater*	1	Adult	DAWE, International Interception	MZ571650	24.3	Negative
*Dermestes*	*Dermestes carnivorus*	1	Adult	DAWE, International Interception	MZ571651	24.4	Negative
*Orphinus*	*Orphinus* sp.	1	Adult	DAWE, International Interception	MZ571652	19.7	Negative
*Reesa*	*Reesa vespulae*	1	Adult	DAWE, International Interception	MZ571653	16.7	Negative
Undetermined^*^	Dermestidae sp. 1	4	Adult	AgVic, Routine Dermestidae Survey	MZ571654	20.4	Negative
Undetermined^*^	Dermestidae sp. 2	1	Adult	AgVic, Routine Dermestidae Survey	MZ571655	19.5	Negative
Undetermined^*^	Dermestidae sp. 3	1	Adult	AgVic, Routine Dermestidae Survey	MZ571656	19.2	Negative
Undetermined^*^	Dermestidae sp. 4	1	Adult	AgVic, Routine Dermestidae Survey	MZ571657	17.5	Negative
Undetermined^*^	Dermestidae sp. 5	4	Adult	AgVic, Routine Dermestidae Survey	MZ571658	17.9	Negative
Undetermined^*^	Dermestidae sp. 6	1	Adult	AgVic, Routine Dermestidae Survey	MZ571659	17.6	Negative
Undetermined^*^	Dermestidae sp. 7	1	Larva	AgVic, Routine Dermestidae Survey	MZ571660	23.3	Negative

Grey shading indicates the target species.

^*^ These Dermestidae specimens represent ‘undetermined’ species currently being identified further by AgVic.

^†^ DNA sequences partial 16S locus.

### 2.2 DNA extraction

We extracted ‘clean’ DNA samples using laboratory‐based Qiagen DNA extraction commercial kits and undertook two simple ‘crude’ extraction methods using HotSHOT and QuickExtract solution, suitable for in‐field use.

Destructive DNA extractions were made from adult Khapra beetles using one or two legs (Table 1) – with a DNeasy Blood and Tissue extraction kit (Qiagen, Hilden, Germany), following the manufacturers protocol, varying only in final elution of 50 μL of AE buffer (instead of the recommended 100 μL). DNA was quantified by NanoDrop ND‐1000 Spectrophotometer (Thermo Fisher, Scoresby, Australia) and stored at −20 °C. This ‘clean’ DNA was used in DNA barcoding and as a positive control in the Khapra and 18S LAMP assay.

DNA from DAWE intercepted Khapra beetle specimens were extracted using a modified non‐destructive protocol. This method uses the same Qiagen extraction kit mentioned earlier with an overnight digest of intact specimens submerged in Proteinase K and ATL buffer at 56 °C and with DNA eluted with 50 μL of AE buffer.^25^ All larval specimens from the greater Melbourne survey had clean DNA extracted using this non‐destructive method, allowing these specimens to be retained as voucher specimens for morphological examination.

For LAMP assays ‘crude’ DNA extracts were prepared from Khapra beetle larvae using a modified HotSHOT protocol ‘HS6’.^26^ Briefly, the intact specimen was placed in 50 μL of premixed 25 mmol L^−1^ sodium hydroxide (NaOH) + TE buffer, pH 8.0 (Invitrogen, Australia) (1:1), pre‐pipetted into each well of a eight‐well Genie strip (OptiGene, UK) and denatured at 95 °C for 5 min in the portable Genie III (OptiGene), followed by > 1 min incubation on ice.^24^ DNA was stored at −20 °C. A second ‘crude’ DNA extraction method was tried using the QuickExtract**™** DNA extraction solution 1.0 (Epicentre, USA). We pipetted 50 μL of QuickExtract (QE) solution into each well of an eight‐well Genie strip holding intact dry beetle larva specimen. The prepared strip was placed in the Genie III machine, used as an incubator, for DNA extraction: 65 °C for 6 min, followed by 2 min at 98 °C.^23^

### 2.3 Development of Khapra beetle LAMP assay

#### 2.3.1 Khapra LAMP primer design

A species‐specific LAMP assay for the detection of Khapra beetle was developed from existing reference DNA sequences of the 16S locus^17^ by targeting primer regions with low intraspecific variation and high interspecific variability in an alignment of *T. granarium* sequences with the 13 most closely related species available.^17^ Six novel LAMP primers were designed manually to target eight DNA regions in the present study, synthesized by Sigma (Australia). For all primers the GC content (%), predicted melting temperature (Tm), and potential secondary structure (hairpins or dimers) were analysed using the integrated DNA technologies (IDTs) online OligoAnalyzer tool (https://sg.idtdna.com/calc/analyzer), using the quantitative polymerase chain reaction (qPCR) parameter sets. Complete sets of LAMP primers were analysed together to detect potential primer dimer interactions using the Thermo Fisher Multiple Primer Analyzer tool (www.thermofisher.com).

#### 2.3.2 18S LAMP primer design

In insects, LAMP primers are usually designed to match DNA sequences from a specific taxon, with amplification providing a positive or negative diagnosis. However, if DNA degradation has occurred, for example through poor preservation of trapped specimens, species‐specific LAMP assays might produce negative results from positive samples due to the poor DNA quality. To address this LAMP primers for 18S (ribosomal) DNA were developed, as earlier, by aligning regions of homology identified manually from diverse invertebrate orders (unpublished data). Six novel LAMP primers targeting eight DNA regions were designed in the present study, as earlier.

#### 2.3.3 Khapra and 18S LAMP primer ratio optimization and assay conditions

LAMP primer ratio optimization and assay conditions for Khapra beetle and 18S were performed following the protocol for Queensland Fruit Fly.^23^ Multiple primer ratios were tested to obtain optimum amplification time and a consistent anneal derivative temperature. For Khapra beetle, Primers F3 and B3 are used at 10 μmol L^−1^ concentration, whilst FIP, BIP, Bloop and Floop are used at 100 μmol L^−1^ concentration. The Khapra primer mix ratio (1:4:2) was prepared by adding 10 μL of each F3 and B3, 4 μL of each FIP and BIP, 2 μL of each Bloop and Floop and 68 μL of water, bringing it to a total volume of 100 μL.

For the 18S LAMP test primer mix ratio (1:6:3) was prepared by adding 10 μL of each F3 and B3, 6 μL of each FIP and BIP, 3 μL of each Bloop and Floop and 62 μL of water, bringing it to a total volume of 100 μL.

The LAMP reaction mixes for both assays (25 μL) were made by adding 10 μL of specific primer mix to 14 μL of Isothermal Master Mix (ISO‐001, OptiGene) and 1 μL of template DNA per well of the Genie strip. The Genie strip of eight wells was made to run six test samples, plus one known Khapra DNA, i.e. a positive control (VAITC 8332d) or a Khapra gBlock, and one no‐template negative control.

Quantification of ‘crude’ (HS6 and QuickExtract) DNA using standard methods, e.g. Nanodrop and Qubit, is considered unreliable. To counter the risk of the Khapra‐specific LAMP assay producing negative results from positive samples we ran the 18S LAMP assay on all DNA extractions to confirm the presence of DNA. Separate Genie strips were run with the same DNA template for Khapra and 18S LAMP assay.

All LAMP assays were run in the Genie III at 65 °C for 25 min followed by an annealing curve analysis from 98 °C to 73 °C with ramping at 0.05 °C s^−1^. The total run time is approximately 35 min. The amplification and anneal derivative curves can be visualized on the Genie III screen to ensure that amplification has occurred as expected. The time of amplification (Time, in minutes) and anneal derivative temperature (Temperature, ^o^C) are recorded from the Results tab displayed by the Genie III. Only with presence of the target DNA the double stranded DNA (dsDNA) is generated allowing a dsDNA intercalating dye to fluoresce. A positive amplification plot shows an ‘S’ shaped sigmoid curve reflecting the increase in fluorescence detected, whilst a negative result stays flat for the duration of LAMP run. Positive results are further confirmed through the annealing step which results in a single product peak with a specific temperature for each LAMP test. LAMP reactions produce amplicons with a specific sequence unique to that target. As such, the amplicon will produce a unique anneal derivative temperature (similar to high resolution melting) which confirms amplification of the target DNA sequence. In the same way, false positive and negative results can easily be distinguished.

All samples which failed to amplify were re‐tested by repeating the reactions, increasing the 65 °C HOLD from 25 to 45 min for both LAMP tests (Khapra and 18S) to ensure no positives were missed due to low quantities of template DNA. We performed student *t*‐tests in Excel to compare the two (Khapra and 18S) LAMP test results.

We recorded the date, Genie III serial number and the run number of each LAMP assay completed on the machine for ease of tracking and downloading results. The run files were transferred and analysed using a personal computer version of the software Genie Explorer version 2.0.7.11, visualized in the blue channel.

### 2.4 Analytic sensitivity of the Khapra LAMP assay compared to real‐time PCR

We made a five‐fold serial dilution (1:5) of clean DNA Khapra beetle extract (specimen VAITC 8332e) using ultrapure water (Invitrogen, Life Technologies, Australia). DNA concentration was quantified with Qubit 2.0 Flourometer (Invitrogen, Life Technologies) following manufacturers protocol. The DNA sample was serially diluted from 10.0 ng μL^−1^ to 1.02 × 10^−6^ ng μL^−1^ (1:1 to 1:9765625). The sensitivity of both LAMP assays was tested using 11 serially diluted DNA samples in the Genie III, following same assay conditions as described earlier. The time of amplification and anneal derivative temperature were recorded for all samples.

Same serial dilution of Khapra beetle DNA was compared for sensitivity using real‐time PCR assay. The primers and probe set (Sigma) and cycling conditions used were as published previously,^17^ the only modification being the extension of annealing temperature time from 30 s to 1 min. Real‐time PCR was performed in QuantStudio**™** 3 Real‐time PCR system (Thermo Fisher Scientific, Scoresby, Australia) in a total volume of 25 μL with technical replicates for each dilution. Each reaction mixture included 12.5 μL Platinum® Quantitative PCR SuperMix‐UDG (Invitrogen), 0.5 μmol L^−1^ of each forward and reverse primers (published 0.2 μmol L^−1^), 0.2 μmol L^−1^ Taqman® probe, 1 μL of template DNA and made up to 25 μL with RNA‐free water. A non‐template control with 1 μL of water instead of DNA was included in each run to control for reagent contamination. The PCR thermal cycling conditions consisted of a two‐step denaturation: 2 min at 50 °C and 10 min at 95 **°**C, followed by 40 cycles of amplification in a two‐step procedure: 95 **°**C for 10 s and 50 **°**C for 1 min. The average Cq value (cycling quantification value) of the 11 dilutions was recorded for comparison with the amplification time from the LAMP assay.

### 2.5 Colorimetric Khapra LAMP assay

We also tested our LAMP assay primers using an alternative, colorimetric LAMP master mix (WarmStart® Colorimetric LAMP 2x master mix (DNA and RNA), New England Biolabs Inc., Ipswich, MA, USA). Briefly, to 12.5 μL of Colorimetric master mix, 2.5 μL of Khapra or 18S LAMP primer mix with describer ratio and 1 μL of known DNA template was added per well, respectively. The first well in each test had target DNA–*T. granarium*, second *T. variabile*, third *Anthrenus verbasci* and fourth no‐template control. The tubes were incubated on a heat block and the colour change was monitored by photographing with a Canon 5D digital SLR camera every 15 min over 2 h. We ran both Khapra and 18S LAMP tests side‐by‐side for comparison, showing a clear timeline for each test.

### 2.6 Evaluation of a gBlock DNA fragment for use as synthetic DNA positive in Khapra LAMP assay

gBlocks™ Gene Fragments, double‐stranded gene fragments > 125 base pair (bp), are commonly synthesized as synthetic positive controls. In our study we designed a gBlock dsDNA fragment (Integrated DNA Technologies, Coralville, IA, USA) modified from Khapra 16S DNA sequences, for use as synthetic DNA positive control for the Khapra LAMP assay. The synthetic fragment consists solely of concatenated LAMP primers separated by ‘ccc’ sections to increase the overall Tm of the fragment.

The starting copy number of Khapra gBlock DNA fragment was calculated using the protocol provided on the IDT website. To evaluate detection sensitivity, a ten‐fold serial dilution (1:10) of the gBlock dsDNA fragment was prepared using TE buffer, pH 8.0 (Invitrogen), as previously outlined.^24^ Synthetic DNA was serially diluted from ~100 million copies down to ~10 copies (10^8^ copies to 10 copies). Sensitivity of the LAMP assay was tested using the serially diluted synthetic DNA in the Genie III, following Khapra LAMP assay conditions (run time increased from 25 to 35 min). Following this run, another LAMP run was done to determine the best dilution value of synthetic DNA for use as positive template in Khapra LAMP assay. The five‐fold serial dilution (VAITC 8332e) (10 to 0.0032 ng μL^−1^) of clean Khapra beetle DNA was used as template for comparison with one million (10^6^) and one hundred thousand copies (10^5^) of synthetic DNA. The amount of Khapra beetle DNA was then equated from the amplification time of 10^6^ copies of synthetic DNA.

## 3 RESULTS

### 3.1 Specimens examined

We examined 119 individual dermestid beetles, 26% of which were intercepted exotic Khapra beetles from two independent international origins, collected almost a year apart, in both larval and adult stages of lifecycle development (Table 1). The majority (87%) of Khapra beetle tested were larvae (Table 2), with some samples being only partial specimens and in poor condition and very difficult to identify morphologically. Other native and exotic non‐Khapra dermestid specimens (preserved adults/larvae) were identified morphologically and through DNA barcoding of a portion of the 16S locus to confirm identifications (Table 1).

**Table 2:** DNA extractions and LAMP (loop‐mediated isothermal amplification) assays from intercepted Khapra beetle specimens

						Khapra LAMP		18S LAMP	
Life stage	Sample	VAITC	Preserved	Extraction type	Extraction method	Time (min)	Anneal derivative (°C)	Time (min)	Anneal derivative (°C)
Adult (leg)	AgVic CHS	8332a	Dry	‘Clean’	Destructive column	Failed	Failed	Failed	Failed
Adult (leg)	AgVic CHS	8332c	Dry	‘Clean’	Destructive column	21.5	77.5	17.2	88.7
Larva (whole)	AgVic CHS	8332d	Ethanol	‘Clean’	Destructive column	16.5	77.8	13.3	89.0
Larva (whole)	AgVic CHS	8332e	Ethanol	‘Clean’	Destructive column	14.0	77.6	13.3	88.9
Larva (whole)	AgVic CHS	8332b	Ethanol	‘Clean’	Non‐destructive column	22.0	77.5	24.2	88.1
Larva (whole)	DA 336226	9619	Dry	‘Clean’	Non‐destructive column	20.2	77.4	13.5	88.7
Larva (whole)	DA 336226	9620	Dry	‘Clean’	Non‐destructive column	19.0	77.3	16.5	88.4
Adult (whole)	DA 336226	9622	Dry	‘Clean’	Non‐destructive column	17.2	77.5	14.0	88.6
Larva (whole)	DA 336226	9623	Dry	‘Clean’	Non‐destructive column	22.5	77.2	18.2	88.3
Pupa (whole)	DA 336226	9624	Dry	‘Clean’	Non‐destructive column	23.3	77.2	24.0	88.3
Larva (whole)	DA 336226	Kh07	Dry	‘Crude’	HS6	20.0	77.6	17.0	88.9
Larva (whole)	DA 336226	Kh08	Dry	‘Crude’	HS6	20.0	77.8	21.5	88.8
Larva (whole)	DA 336226	Kh09	Dry	‘Crude’	HS6	23.3	77.4	17.5	88.9
Larva (whole)	DA 335997	Kh04	Ethanol	‘Clean’	Non‐destructive column	18.3	77.7	15.3	88.7
Larva (whole)	DA 335997	Kh05	Ethanol	‘Clean’	Non‐destructive column	Failed	Failed	Failed	Failed
Larva (whole)	DA 335997	Kh06	Ethanol	‘Clean’	Non‐destructive column	24.2	77.3	19.0	88.8
Larva (whole)	DA 335997	Kh01	Ethanol	‘Crude’	HS6	18.3	78.2	18.0	88.9
Larva (whole)	DA 335997	Kh02	Ethanol	‘Crude’	HS6	16.0	78.3	15.3	88.8
Larva (whole)	DA 335997	Kh03	Ethanol	‘Crude’	HS6	Failed	Failed	Failed	Failed
Larva (whole)	DA 335997	Kh10	Ethanol	‘Crude’	HS6	Failed	Failed	Failed	Failed
Larva (whole)	DA 335997	Kh11	Ethanol	‘Crude’	HS6	Failed	Failed	Failed	Failed
Larva (whole)	DA 335997	Kh12	Ethanol	‘Crude’	HS6	Failed	Failed	Failed	Failed
Larva (whole)	DA 336226	Kh13	Dry	‘Crude’	QuickExtract™	16.3	78.1	14.5	88.6
Larva (whole)	DA 336226	Kh14	Dry	‘Crude’	QuickExtract™	19.2	78.4	12.3	88.5
Larva (whole)	DA 336226	Kh15	Dry	‘Crude’	QuickExtract™	18.3	78.2	15.5	88.6
Larva (whole)	DA 335997	Kh16	Ethanol	‘Crude’	QuickExtract™	22.5	77.9	21.3	88.7
Larva (whole)	DA 335997	Kh17	Ethanol	‘Crude’	QuickExtract™	22.5	77.3	21.5	88.0
Larva (whole)	DA 335997	Kh18	Ethanol	‘Crude’	QuickExtract™	23.5	77.2	24.0	88.4
Larva (whole)	DA 335997	Kh19	Ethanol	‘Crude’	QuickExtract™	24.2	77.1	20.3	88.7
Larva (whole)	DA 335997	Kh20	Ethanol	‘Crude’	QuickExtract™	Failed	Failed	Failed	Failed
Larva (whole)	DA 335997	Kh21	Ethanol	‘Crude’	QuickExtract™	24.2	77.1	17.5	88.5
					Average:	20.3	77.6	17.7	88.6
					Minimum:	14.0	77.1	12.3	88.0
					Maximum:	24.2	78.4	24.2	89.0

Each line is a different individual insect.

### 3.2 Khapra and 18S LAMP assay

**Figure 1:**
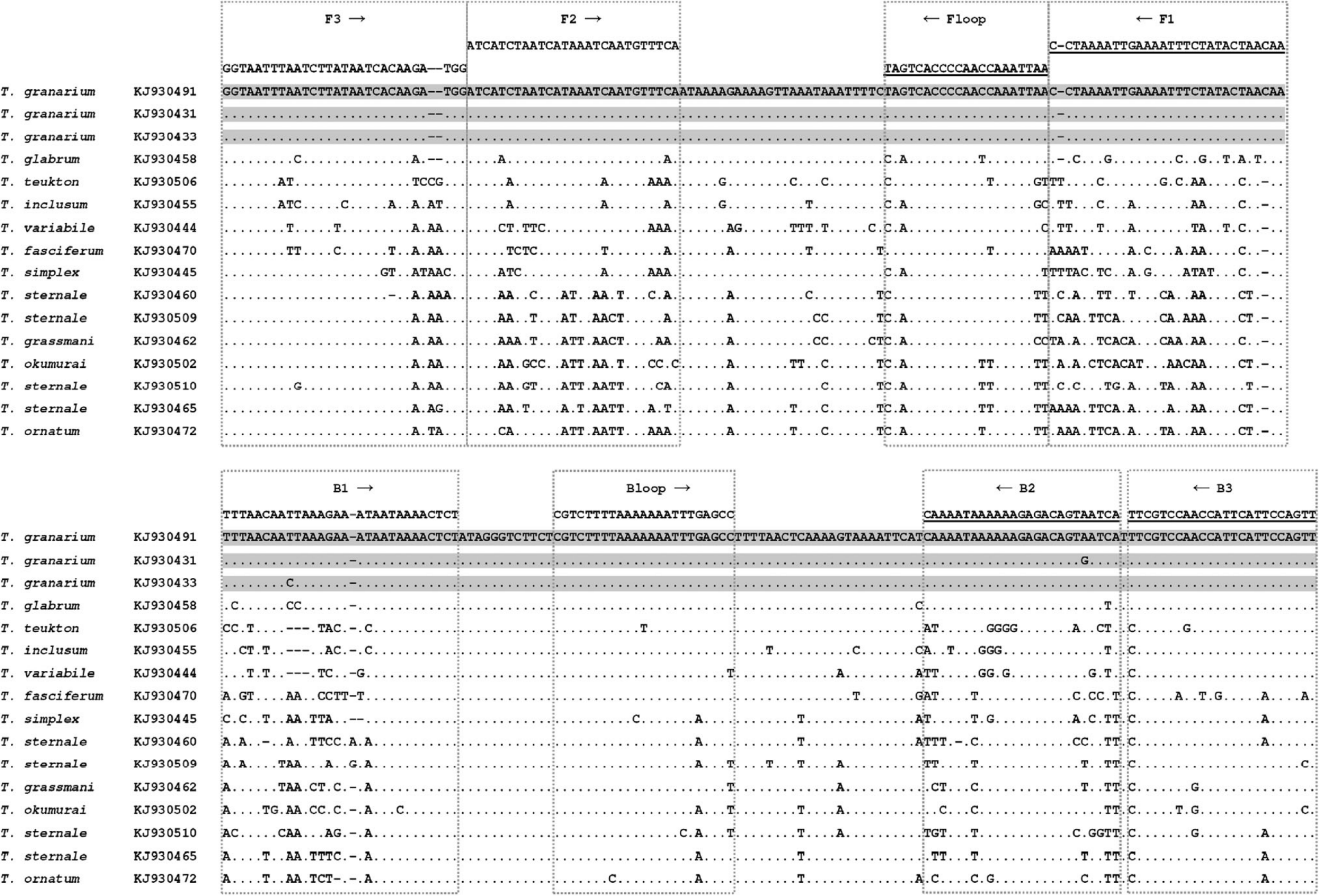
16S DNA sequence alignment (274 bp) showing Khapra LAMP primers. Sequences of three *Trogoderma granarium* individuals (grey shading) and other closely related *Trogoderma* species. GenBank accession numbers are shown, from Olson *et al*.^17^ Reverse primers are underlined; FIP (5′–3′) is made by combining F1 (reverse compliment) and F2; BIP (5′–3′) is made by combining B1 and B2 (reverse compliment).

Novel LAMP primers (Table 3) were developed to target a 274 bp portion of the Khapra beetle 16S locus (Fig. 1). This region is highly variable and has been previously characterized in numerous Dermestidae species, ^17^ with Khapra beetle being 6.5% to 7.5% divergent from the most similar species *T. glabrum* for this the 16S locus (Fig. 1). The Khapra LAMP assay consists of six primers, including forward and backward loop primers, which were found to result in more rapid amplification times. The optimal primer ratio (F3/B3:FIP/BIP:Floop/Bloop) was determined to be 1:4:2 and final concentrations of 0.4, 1.6, and 0.8 μmol L^−1^ for the F3/B3, FIP/BIP and Floop/Bloop primers, respectively. In this study we employed an 18S locus control LAMP assay to test for the presence of beetle DNA. The 290 bp 18S LAMP region contains six primers (Table 3). The optimal primer ratio (F3/B3:FIP/BIP:Floop/Bloop) was determined to be 1:6:3 with final concentrations of 0.4, 2.4, and 1.2 μmol L^−1^ for the F3/B3, FIP/BIP and Floop/Bloop primers, respectively.

**Table 3:** LAMP (loop‐mediated isothermal amplification) primer and amplicon sequences (gBlock) and parameters

LAMP primer or amplicon	Sequence 5′–3′	Primer length (bp)	Predicted Tm, annealing temperature (°C)	Degeneracy of primer (fold)
Khapra_gBlock fragment	cccGGTAATTTAATCTTATAATCACAAGATGGcccATCATCTAATCATAAATCAATGTTTCAcccTAGTCACCCCAACCAAATTAAcccCCTAAAATTGAAAATTTCTATACTAACAAcccTTTAACAATTAAAGAAATAATAAAACTCTcccCGTCTTTTAAAAAAATTTGAGCCcccCAAAATAAAAAAGAGACAGTAATCAcccTTCGTCCAACCATTCATTCCAGTTccc	234	N/A	None
Khapra_F3	GGTAATTTAATCTTATAATCACAAGATGG	29	60.9	None
Khapra_B3	AACTGGAATGAATGGTTGGACGAA	24	69.2	None
Khapra_FIP	TTGTTAGTATAGAAATTTTCAATTTTAGGATCATCTAATCATAAATCAATGTTTCA	56	73.8	None
Khapra_BIP	TTTAACAATTAAAGAAATAATAAAACTCTTGATTACTGTCTCTTTTTTATTTTG	54	70.7	None
Khapra_Floop	TTAATTTGGTTGGGGTGACTA	21	60.7	None
Khapra_Bloop	CGTCTTTTAAAAAAATTTGAGCC	23	61.6	None
*Insect 18S*				
18S_F3	AGAGGTGAAATTCTTGGATCGTC	23	64.3	None
18S_B3	CCCGTGTTGAGTCAAATTAAG	21	61.0	None
18S_FIP	GGTTAGAACTAGGGCGGTATCKAAGCGAAAGCATTTGCCA	40	80.1	2
18S‐BIP	TCCGGGGGAAGTATGRTTGCAAAGGCTCCACTCCTGGTGGT	41	88.9	2
18S_Floop	GCCTTCGAACCTCTAACTTTC	21	60.8	None
18S_Bloop	TGAAACTTAAAGGAATTGACGGAA	24	64.4	None

The F2 and B2 primer regions of FIP and BIP are underlined. Lowercase letters in the gBlock indicate extra c's added between LAMP primer sites to increase the overall melting temperature (Tm) of the amplicon.

### 3.3 Performance and specificity of the Khapra and 18S LAMP assays

**Figure 2:**
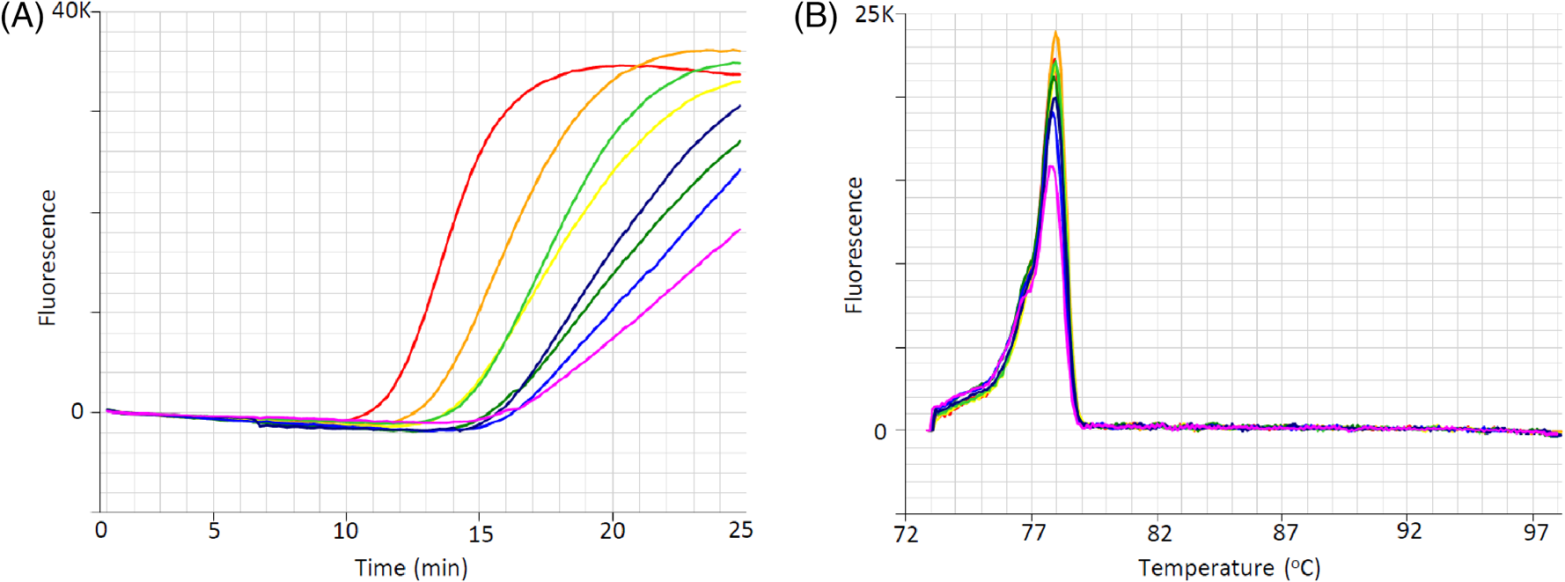
Khapra LAMP assay results for a DNA serial dilution of *Trogoderma granarium* VAITC8332d. (a) Amplification profile, with positive samples amplifying in < 20 min. (b) Anneal derivative of LAMP amplicons, with ananneal derivative temperature of approximately 77.7 °C.

We tested the specificity of the new LAMP assays by screening a large panel of Dermestidae beetles, including 23 ‘non‐target’ dermestid species and numerous Khapra beetle samples (Table 1). The Khapra LAMP assay produced amplification of the target on average at 19.8 min (range: 14.0–24.2 min), with an anneal derivative temperature of 77.7 °C (range: 77.1–78.4 °C), with any amplification under ≤ 25 min considered positive (Fig. 2). The assay was found to be very specific with only the target species, *T. granarium*, found to amplify. The 18S LAMP control assay produced amplification of the present DNA on average at 17.7 min (range: 12.3–24.2 min), with an anneal derivative temperature of 88 °C (range: 88.0–89.0 °C), with any amplification under ≤ 25 min considered positive (Fig. 2). The assay was found to be very specific with only the samples with sufficient good quality DNA found to amplify.

All non‐target Dermestidae species, including the most closely related *T. glabrum* (Fig. 1), did not amplify (Table 1). During the local survey we found several individuals of other Dermestidae species which did not match Khapra beetle molecularly, being 17.5% to 23.3% divergent (Table 1), which remained morphologically undetermined due to ambiguity within the Dermestidae family. The use of non‐destructive DNA extraction methods on specimens retained intact vouchers which were available for morphological identification, supported by the 16S barcoding results (Table 1) which verified these specimens were not Khapra beetles.

### 3.4 Performance of DNA extractions in Khapra and 18S LAMP assays

**Figure 3:**
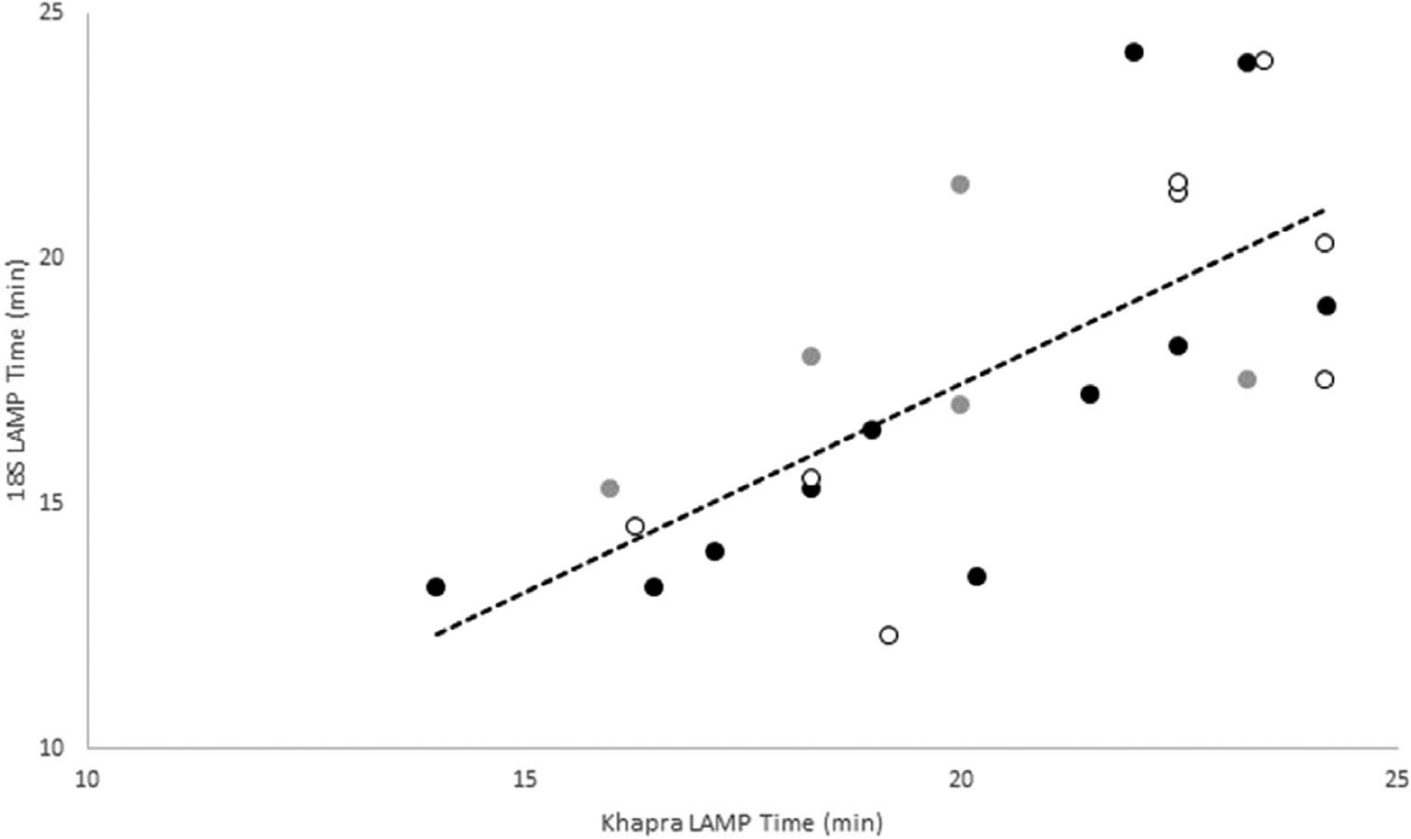
Comparison of amplification times using the Khapra LAMP and 18S LAMP assays on intercepted *Trogoderma granarium* specimens (Table 2). DNA extractions (from individual insects): black dots Qiagen columns (*n* = 11), grey dots HS6 (*n* = 5), white dots QuickExtract (*n* = 8) (Table 2). Linear regression line *R* ^2^ = 0.50.

The 31 Khapra beetle DNA extractions (Table 4) prepared using four different extraction methods were tested with both Khapra and 18S LAMP assays. The approximate quantity (concentration) of extracted Khapra DNA, compared through the 18S LAMP assay, was moderately correlated with amplification success and time for the Khapra assay (Fig. 3). However, specimen preservation was found to have the largest effect on amplification success, with 33% of specimens preserved in diluted ethanol (70%) failing to amplify for both LAMP assays (Table 4) compared with less than 8% of dry specimens across all extraction methods. The method of DNA extraction (clean versus crude) also had a large effect, with less than 16% of clean samples failing to amplify compared with more than 27% of crude DNA extractions. Specimen preservation effects on amplification time and anneal derivative temperature of the two LAMP tests was examined. We found that there was no difference between dry or ethanol preserved specimens for amplification time (LAMP Khapra: *t*_**0.05(2)22**_ = 0.821; LAMP 18S: *t*_**0.05(2)22**_ = 0.237) and same was true when clean versus crude DNA extractions were compared (LAMP Khapra: *t*_**0.05(2)22**_ = 0.549; LAMP 18S: *t*_**0.05(2)22**_ = 0.497). There was also no effect on anneal derivative temperature between the two LAMP tests around the mean. The average anneal derivative temperature for Khapra LAMP was 77.7 ± 0.7 °C, and 88.8 ± 1.0 °C for 18S LAMP (Tables 2 and 4).

**Table 4:** Summary of results for all Khapra beetles tested by various DNA extraction methods, from dry or ethanol preserved samples

					Khapra LAMP		18S LAMP	
					Time (min)	Temperature (°C)	Time (min)	Temperature (°C)
DNA extraction method	Destructive (D), non‐destructive (ND)	Extraction type	Preservation method	*n* (failed)	mean ± SD	mean ± SD	mean ± SD	mean ± SD
Qiagen column	D	‘Clean’	Dry	2 (1)	21.5 ± 0	77.5 ± 0	17.2 ± 0	88.7 ± 0
Qiagen column	D	‘Clean’	Ethanol	2 (0)	15.2 ± 1.3	77.7 ± 0	13.3 ± 0	88.9 ± 0
Qiagen column	ND	‘Clean’	Dry	5 (0)	20.4 ± 2.2	77.3 ± 0.1	17.3 ± 3.8	88.5 ± 0.2
Qiagen column	ND	‘Clean’	Ethanol	4 (1)	21.5 ± 2.4	77.5 ± 0.2	19.5 ± 0.3	88.5 ± 0.3
HS6	ND	‘Crude’	Dry	3 (0)	21.1 ± 1.5	77.6 ± 0.2	18.6 ± 2.0	88.7 ± 0
HS6	ND	‘Crude’	Ethanol	6 (4)	17.5 ± 1.1	78.3 ± 0	16.6 ± 1.3	88.8 ± 0
QuickExtract™	ND	‘Crude’	Dry	3 (0)	17.9 ± 2.4	78.2 ± 0.4	14.1 ± 1.3	88.6 ± 0
QuickExtract™	ND	‘Crude’	Ethanol	6 (1)	23.6 ± 0.8	77.2 ± 0.3	20.8 ± 2.1	88.5 ± 0.2
			All methods	31 (7)	19.8 ± 2.6	77.7 ± 0.4	17.2 ± 2.4	88.7 ± 0.2

Individual specimen results are in Table 2. SD, standard deviation.

### 3.5 Sensitivity of LAMP and real‐time PCR assays

**Figure 4:**
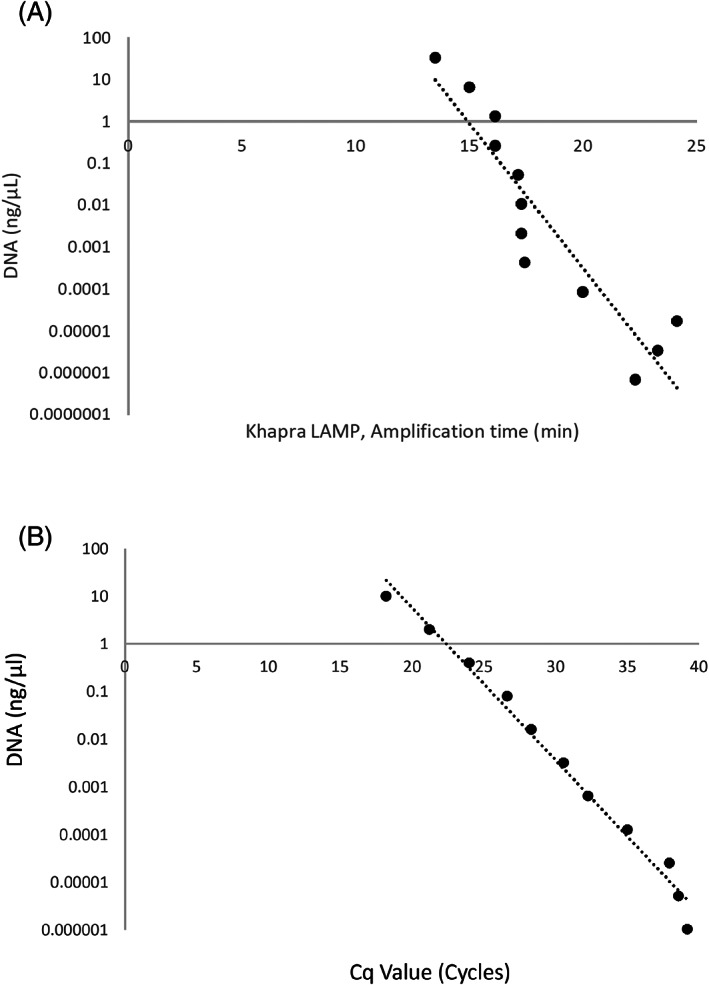
Comparison of Khapra LAMP and Khapra real‐time qPCR assays on *Trogoderma granarium* VAITC8332e DNA dilution series (a) Khapra LAMP, exponential regression line, *R* ^2^ = 0.86. (b) Real‐time qPCR exponential regression line, *R* ^2^ = 0.99.

The sensitivity of the Khapra LAMP assay was compared with an existing laboratory based Khapra real‐time PCR test. The real‐time PCR test produced reliable amplification, using the modified conditions in our study. Both LAMP and real‐time PCR performed similarly on serial DNA dilutions down to 1.02 × 10^−6^ ng μL^−1^, with positive amplification from high to very low DNA concentrations (Fig. 4). At the lowest DNA concentrations, of 1.02 × 10^**−**6^ ng μL^−1^, LAMP amplification time was < 25 min (Fig. 4), compared with an average Cq value of 39, which was our threshold for positive samples using real‐time PCR (Fig. 4). A strong relationship between DNA concentration and LAMP amplification (*R* ^2^ = 0.86) and real‐time PCR amplification (*R* ^2^ = 0.99) was observed. As in real‐time PCR, amplification in the LAMP assay was found to become slower in a predictable manner as DNA template concentrations were reduced, showing a strong relationship between increased amplification times and decreasing DNA concentrations.

### 3.6 Colorimetric LAMP detection

**Figure 5:**
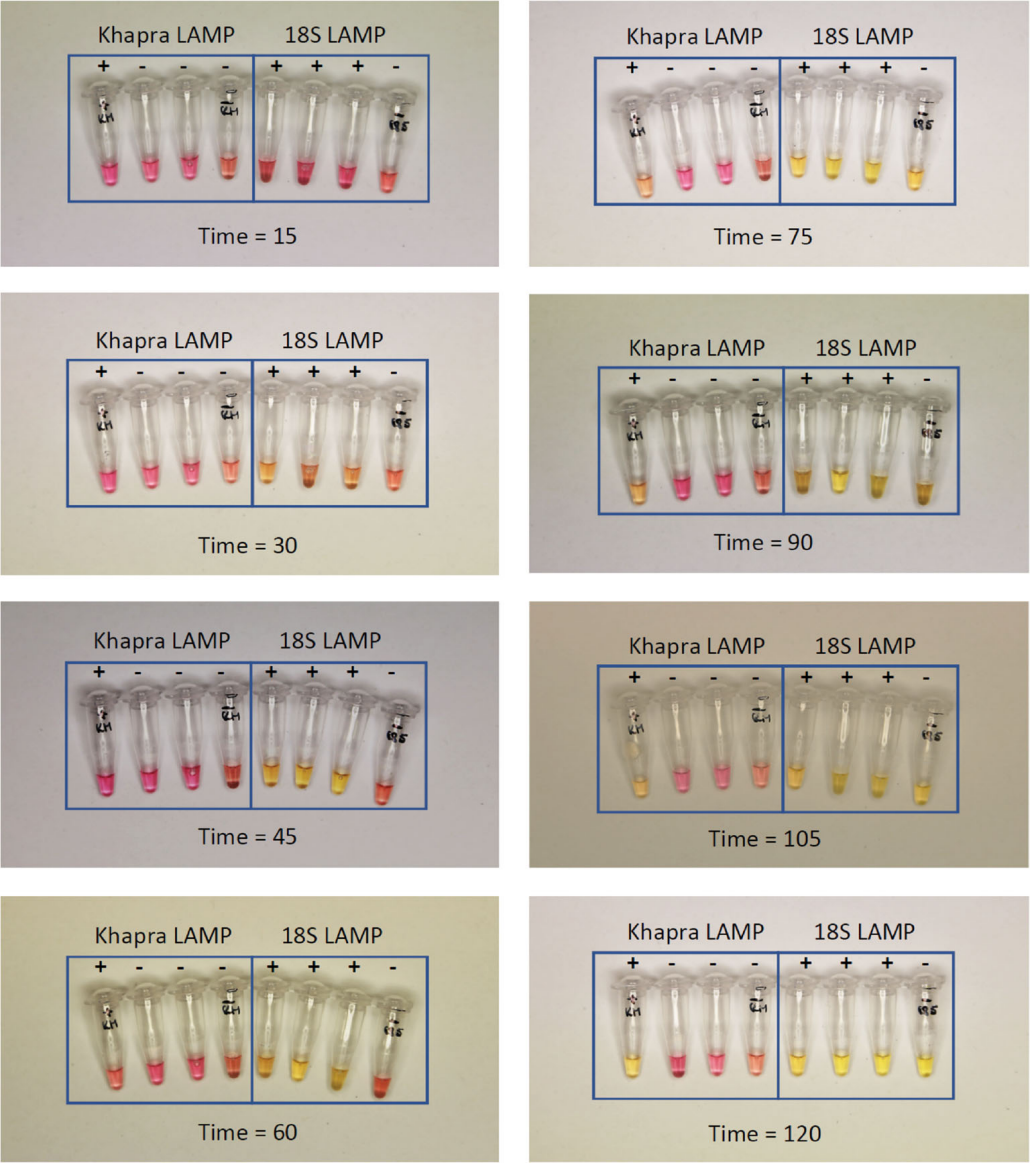
Time‐series of Khapra LAMP (left) and 18S LAMP (right) using ‘colorimetric master mix. Two‐hour total amplification time shown in increments of 15 min. ‘+’ indicates the sample is expected to be positive for the LAMP assay, ‘−’ indicates the sample is expected to be negative for the LAMP assay. Samples: (1) *Trogoderma granarium* VAITC8332d, (2) *Trogoderma variabile* VAITC9217, (3) *Anthrenus versicolor* VAITC9112, (4) no‐template negative control.

We developed a complimentary, simple colorimetric LAMP test which can be used in the field conditions if a portable real‐time fluorometer machine is not available. The LAMP colorimetric reactions can be performed on a simple heating block (at 65 **°**C) with the colour in the wells changing from pink to yellow indicating positive samples. Amplification using colorimetric master mix was found to take significantly longer compared with use of standard OptiGene reagents. The 18S LAMP assay was found to produce positive results after 1‐h incubation whilst Khapra LAMP assay took 1½ to 2 h for results to be observed (Fig. 5). If these times are not respected and reactions are left for longer at 65 °C, all wells (including the negative control) eventually changed colour to yellow (indicating positive samples) losing meaningful diagnostic information. Out of four species tested in colorimetric assay for the Khapra LAMP assay only *T. granarium* produced a positive colour change, further showing the robustness of our new assay (Fig. 5). Both LAMP assays were shown to perform well on either a portable real‐time fluorometer or using a colorimetric master mix.

### 3.7 Detection sensitivity of gBlock DNA fragment

**Figure 6:**
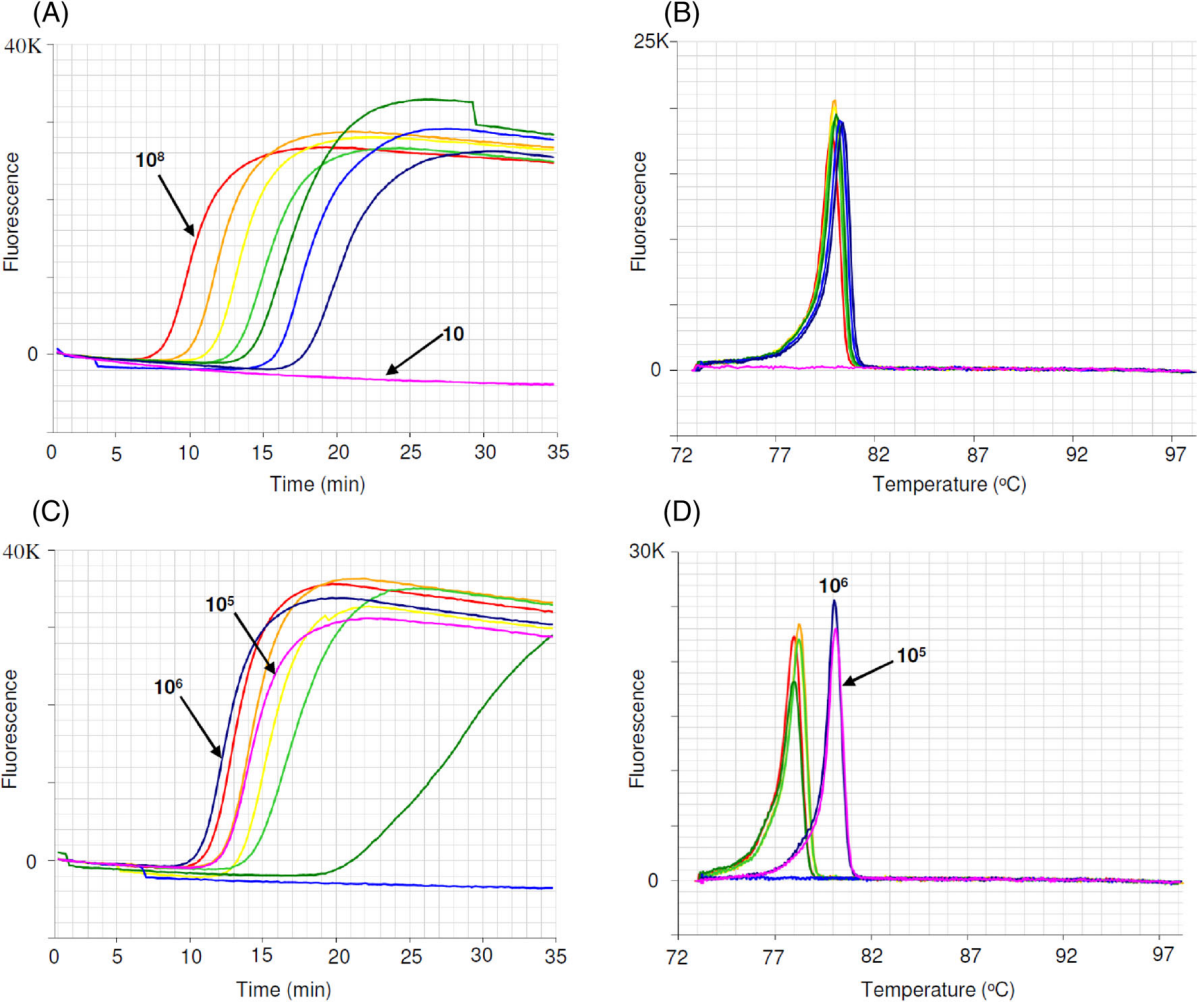
Detection sensitivity of Khapra gBlock dsDNA amplicons (upper), evaluating amount of Khapra DNA with synthetic DNA (lower). (a) Amplification profile with templates ranging from 10^8^ to 10 copies at ten‐fold dilution (pink, no amplification). (b) Anneal derivative of LAMP amplicons, with an anneal derivative of ~80 °C. (c) Amplification profile of five‐fold dilution of Khapra DNA (VAITC8332e) ranging from 10 to 1.0^−4^ ng μL^−1^ and synthetic DNA (10^6^ copies, blue and 10^5^ copies, pink). (d) Anneal derivative of LAMP amplicons showing two peaks, ~78 °C for khapra DNA and ~80 °C for synthetic DNA (blue and pink).

The detection sensitivity of the Khapra 234 bp gBlock dsDNA fragment (Table 3) was evaluated for templates ranging from ~100 million copies down to ~10 copies at ten‐fold dilution in LAMP reactions (Fig. 6). The detection level was quite sensitive, detecting as low as ~100 copies within 25 min (Fig. 6(a)) with an anneal derivative of 80 °C (Fig. 6(b)). One million and one hundred thousand copies of gBlock fragments were compared to five‐fold dilution of Khapra beetle DNA ranging from 10 to 0.0032 ng μL^−1^ (Fig. 6(c)). From the amplification profile one million copies (10^6^) of synthetic DNA equates to ~10 ng μL^−1^ of Khapra beetle DNA. The anneal derivative of LAMP amplicons in this run shows two peaks, 78 °C for Khapra beetle DNA and 80 °C for the synthetic DNA fragment (Fig. 6(d)).

## 4 DISCUSSION

In our study we developed a novel LAMP test for in‐field identification of Khapra beetle – *T. granarium* Everts, a major worldwide pest of stored products. We utilized existing sequence variation of 16S locus from 13 closely related *Trogoderma* species^17^ to design new Khapra specific LAMP primers. The optimized assay proved to be rapid and robust, with amplification in under 25 min, and very specific, with only *T. granarium* producing positive amplification. Whilst the number of species closely related to Khapra beetle available to be tested in this study was limited due to the majority of *Trogoderma* species being exotic to Australia, the most closely related species, *T. glabrum* was obtained from an international interception and tested, as were four more distant *Trogoderma* species which all failed to amplify using the Khapra LAMP test. Genetic variation of 16S DNA sequence variation within *T. granarium*, from multiple geographic sources worldwide, has previously been characterized,^17^ with intraspecific variation found to be very low (< 0.5%), compared with large differences apparent between Dermestidae species. The two sample groups of Khapra included in the current study matched known 16S haplotypes.^17^ No amplification was observed in a further 17 species, representing at least additional six Dermestidae genera. In our study some taxa could not be completely identified morphologically, as many Australian species are yet to be described morphologically or sequenced to allow identification using DNA barcodes; our understanding of the true morphology of Dermestidae in Australia is not complete, with taxonomic revisions currently being undertaken (Adam Slipinski, pers. com.).

Similar to real time PCR, LAMP performed well, proving to be sensitive to very low DNA levels. The Khapra LAMP assay was found to be effective on both adult and larval stages of the Khapra beetle. Often, it is difficult to identify intercepted beetles in the sample as either larval or adult stages can be damaged and in poor condition, therefore this LAMP assay not only provides rapid identification but also gives confidence where morphological work would be impossible or demand a high‐level of entomological expertise. Although there are other Khapra beetle molecular identification tests available, they require complex laboratory methods and longer lead time to results, making this new Khapra LAMP assay invaluable in the field or laboratory environments for confident and expedient Khapra beetle identification information.

In addition to using a portable real‐time fluorometer for LAMP amplification we tested an alternative colorimetric LAMP method which would enable the use of LAMP technology in environments where highly specialized equipment is not available. Both systems showed our new Khapra LAMP assay to be accurate and reliable. We also designed and optimized a synthetic DNA positive control (gBlock) for use in Khapra LAMP assay. The synthetic DNA is beneficial in: (i) providing a consistent control to allow tracking of the performance of LAMP assays across runs, (ii) providing a relatively high amount of control DNA compared with DNA extractions from natural Khapra beetle specimens which can produce low DNA yields due to their small size, and (iii) providing confidence that positive amplification of samples is not due to contamination as synthetic DNA has a different annealing temperature compared to Khapra beetle DNA.

Khapra beetles can rapidly build up into large numbers if not detected in a timely manner, thus providing many specimens suitable for LAMP testing but often, many are degraded. We have shown that it is useful to test DNA quality of specimens (using the 18S LAMP control assay) to know whether a sample was potentially capable of amplification (the presence of good quality and sufficient quantity of DNA) for the Khapra specific LAMP assay, thus avoiding false negative results. The ability to run the two LAMP assays using the same protocol on a portable real‐time fluorometer enables the user to simultaneously test for Khapra beetle and to test DNA presence/quality in the same run, thus saving time. With specimens in poor condition that are less likely to yield sufficient good quality DNA, we would advise running the amplification step of the LAMP assay for longer, 45 min amplification (rather than the standard 25 min), as we have demonstrated that low amounts of DNA template slow down amplification times creating possibility of false negative. However, some samples may be degraded to the point where amplification is no longer possible. Often the only trace detected during surveillance may be the larval/pupal exuviae (moulted skins), which are less likely to contain usable quantities of DNA. In the current study we tested a small number of exuviae samples (*n* = 6), with extended amplification times without success. However, the use of alkaline lysis solution (0.3 mol L^−1^ potassium hydroxide) incubation at 95 °C for 5 min for DNA extractions and an alternative LAMP Isothermal Master Mix (ISO‐004, OptiGene), is an alternative approach which has been shown to regularly produce amplification from Khapra exuviae using our new LAMP assay (L. Watson and K. Sparks, unpublished data).

We have shown here that it is preferable for suspect beetle specimens to be stored dry rather than preserved in 70% ethanol, as the presence of water greatly affects the quality of the extracted DNA. Other environmental conditions including heat and humidity are also likely to greatly degrade the quality of specimens. Such negatively affected specimens can prove difficult to amplify.

In the current study we tested three non‐destructive DNA extraction protocols including commercial kits and crude methods, all of which enabled us to retain intact specimens for further morphological work and species identification. The Khapra LAMP test performed equally well with crude DNA extracts where sample preservation method was critical for assay success. The use of crude non‐destructive DNA extraction methods for in‐field use further provides intact physical voucher specimen as evidence for the presence/absence of the target species, identification of which can be subsequently confirmed by morphological means. This latter point is critical given the trade implications of the establishment of Khapra beetle in a country.^10^

The new assays we have developed and optimized is a portable molecular method that is easy to use both in laboratory and in‐field situations, thus increasing available tools for rapid identification of Khapra beetle, a pest of worldwide biosecurity significance. This Khapra LAMP test has already been adopted in the laboratory as a support to surveillance in Victoria, Australia, for establishing Khapra beetle area freedom following an incursion, thus providing an additional level of confidence to a team of entomology diagnosticians (unpublished data). With the Khapra LAMP test providing conclusive results in under an hour this technology significantly shortens the identification times providing real‐time support to the diagnosticians. The speed of result delivery has further beneficial effects on the decision making that can influence immediate actions with consequences for biosecurity. Further application of this Khapra LAMP assay could be in the sphere of international trade helping to clear goods for transport in a rapid manner thus saving time and money to the grain industry locally and worldwide.

## CONFLICTS OF INTEREST

None to declare.

## AUTHOR CONTRIBUTIONS

This manuscript was drafted by Lea Rako, Mark J Blacket and Arati Agarwal, with contributions from all authors. The identifications of Dermestidae species were sourced and confirmed by Linda Semeraro, Adam Broadley and Lea Rako. The LAMP primers for the assay were designed by MJB. Laboratory work and DNA sequence analyses were performed by Lea Rako, Mark J Blacket and Arati Agarwal. Brendan C Rodoni planned, obtained funding, and assisted with implementation of this study.

## SUPPORTING INFORMATION

The Dermestidae DNA sequences generated in this study have been submitted to GenBank. Accession numbers MZ571636–MZ571660.

## CONSENT FOR PUBLICATION

DAWE and the authors provide consent for publication of this manuscript.
